# Biological Evaluation of the Activity of Some Benzimidazole-4,7-dione Derivatives

**DOI:** 10.3390/molecules191015361

**Published:** 2014-09-26

**Authors:** Katarzyna Błaszczak-Świątkiewicz, Elżbieta Mikiciuk-Olasik

**Affiliations:** Department of Pharmaceutical Chemistry and Drug Analysis, Medical University, Muszynskiego 1, Lodz 90-151, Poland; E-Mail: elzbieta.mikiciuk-olasik@umed.lodz.pl

**Keywords:** anticancer activity, benzimidazole-4,7-dione derivatives, bioreductive prodrugs, bioreductive agents, hypoxia

## Abstract

The study presented here is a follow up of the authors’ interest in the approach to selective and cytotoxic bioreductive anticancer prodrugs. The current work is devoted to explore both the biological activity of some previously obtained compounds and the search for an explanation of their target(s) in hypoxic pathways. In this work the biological activity of some benzimidazole-4,7-diones was evaluated. These compounds were examined as potential bioreductive agents specific for the hypoxic environment found in tumor cells. The main aim was concerned with establishing their cytotoxic properties by using proliferation, apoptosis and DNA destruction tests on selected tumor cells. Their cytotoxic effects on two tumor cell lines (human lung adenocarcinoma A549 cells line and human malignant melanoma WM115) was compared by means of a WST-1 test. Next, the mode of cytotoxicity behind the selected tumor cells’ death was determined by the caspase 3/7 test. The last point referred to the DNA destruction of A549 and WM115 cells and the *in situ* DNA Assay Kit test was applied. The cytotoxic tests confirmed their activity against the tumor cells and target hypoxia (compounds **2b**, **2a**, **2d**). The screening test of the caspase-dependent apoptosis proved that the exposure of the tested tumor cells in hypoxia to these benzimidazole-4,7-diones promoted the apoptotic cell death. Additionally, the DNA damage test established that benzimidazole-4,7-diones can be potential hypoxia-selective agents for tumor cells, especially compound **2b**. All results classify the tested benzimidazole-4,7-diones as promising, lead molecules and provide a rationale for further molecular studies to explain their usefulness as potential inhibitors of the hypoxia-inducible factor 1 (HIF1).

## 1. Introduction

A new approach in the anticancer therapy concerns the use of bioreductive agents as prodrugs specific for tumor cells with very low oxygen concentration. This hypoxia is a characteristic target for anticancer treatment. Prodrugs activated by hypoxia and hypoxia-selective gene therapy are the two main strategies being intensively developed in this context [1]. Nitroaromatic groups, quinones, aromatic *N*-oxides and aliphatic *N*-oxides are some of the chemical groups that have the potential to be metabolized by enzymatic reduction under hypoxic conditions, and due to this fact they represent the basis for designing some new bioreductive prodrugs [2] (Table 1).

**Table 1:** Characteristics of some bioreductive prodrugs [2].

Prodrug	Chemical Structure	Chemical Class	Mechanism of Action *	Mechanism of Cytotoxicity	One-Electron Reductases	Two-Electron Reductases	K_O2_ (µM)
TPZ	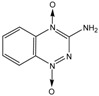	Aromatic *N*-oxide	1, 3 [R^.^] ***	Complex DNA damage	CYPOR ** iNOS **	NQO1 **	±1
SN30000	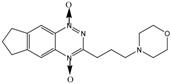	Aromatic *N*-oxide	1, 3 [R^.^] ***	Complex DNA damage	CYPOR **	----	±1
AQ4N	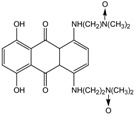	Aliphatic *N*-oxide	2, 5 [Y] ***	Topoisomerase II inhibition	iNOS **	CYP3A4 ** CYP2S1 **	----
EP-0152R plus CB1954	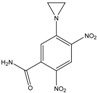	Nitro	1/2, 4, 5, 6 [Y, Z] ***	DNA interstrand crosslink	CYPOR ** iNOS **	NQO1 ** NQO2 **	----
EO9	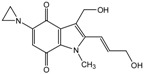	Quinone	1, 4 [X, Y] ***	DNA interstrand crosslink	CYPOR **	NQO1 **	----

* Reaction numbers: 1: one electron reduction generates a prodrug radical; 2: fragmentation of the prodrug radical generates radical R. and cytotoxin D; 3: one electron reduction of the prodrug radical; 4 and 5: subsequent reduction of the two electron reduction produces X; 6: two-electron reduction of the prodrug generates product X; ** CYPOR-NADPH–cytochrome P450 reductase, iNOS-inducible nitric oxide synthase, NQO-NAD(P)H dehydrogenase [quinone], CYP-cytochrome P 450; *** Active cytotoxins (R·, X, Y, Z). All abbreviations refer to reference [2] Figure 2A.

The compounds such as CB1954, PR-104 or TH-302 represent the nitroaromatic group class and they are known as hypoxia-activated prodrugs [2]. The one-electron adduct (the nitro-radical anion) can be scavenged by molecular oxygen, restricting activation in hypoxic cells. Particularly, these nitro drugs are effectively converted by the *Escherichia coli* (*E. coli*) enzyme nitroimidazole reductase (NTR) into the cytotoxic species rather than by human isoforms of NAD(P)H dehydrogenase quinone 1 (NQO1). Therefore, a virus directed enzyme-prodrug therapy (VDEPT) approach may offer tumour specific cell killing [3]. The main mechanism of cytotoxicity of nitro agents is DNA interstrand crosslinking [2]. The potential for using quinones as bioreductive prodrugs emerged with the discovery that the anticancer antibiotic mitomycin C causes DNA-crosslinking which is activated by reducing its indoloquinone moiety [2]. As activation of this drug is inhibited by an oxidizing environment, it was thought that mitomycin C would have selective toxicity for hypoxic solid tumours, and potently suppress their growth [4]. However, its hypoxic selectivity is limited and does not represent a useful basis for it. Porfiromycin, its analogue, shows a greater hypoxic selectivity [5]. The indoloquinone EO9 is another quinone-based hypoxia-activated prodrug. Although this drug did not show clinical activity as a single agent in phase II studies, it is still currently under development. The aromatic *N*-oxide class is best represented by tirapazamine (TPZ), which is the most clinically useful hypoxia-activated prodrug discovered to date. TPZ shows a high (100 to 200 fold) hypoxic selectivity in cell suspension cultures, but its metabolism limits its diffusion through tissue to the non-diffusible, radical species. However, this drug tends to be selectively cytotoxic to the hypoxic fraction of cells in a few animal tumours *in vivo*. The fact that the TPZ radical is much more cytotoxic than the superoxide radical leads to this specific hypoxic cytotoxicity [5,6,7]. It has been suggested that TPZ has a dual mechanism of action, both generating DNA radicals and, also oxidizing these radicals to form DNA breaks [8]. It was clearly shown that TPZ enhances the antitumor efficacy of some antitumor drugs, namely cisplatin [9]. This interaction may be the reason for a delay in repairing the cisplatin-induced interstrand cross-links in cells which are exposed to TPZ under hypoxia before cisplatin exposure [6,10]. As far as the aliphatic *N*-oxides are concerned, hypoxic selectivity is much more due to the result of the inhibition of two-electron reductases by oxygen, rather than redox cycling. The best example of this class is AQ4N, in which the inhibition by oxygen of the bioreduction of aliphatic *N*-oxides to the corresponding tertiary amines is the basis for its mechanism of action. In addition, AQ4N is the only bioreductive prodrug topoisomerase II inhibitor to enter clinical trials [2,5,11]. Moreover, derivatives which contain the benzimidazole ring act as human DNA topoisomerase I [12,13,14] inhibitors and are expected to possess anticancer properties [15] and affinity which is selective for cells under hypoxic conditions [12]. In this context, benzimidazole-4,7-diones derivatives play an important role as quinones are known to have noticeable antitumor activity [16,17,18]. Additionally, benzimidazolediones are dominant as intercalators and inhibitors of topoisomerase due to their ability to inhibit hypoxia-inducible factor 1 (HIF1). Developing this thesis the authors planned a variety of tests with benzimidazolediones in order to confirm their potential effectiveness in intercalation in the DNA chain. Because of this, their cytotoxicity towards the two selected cell lines was determined. Following that, their ability to act over the programmed cell death (apoptosis) and their influence on the damage of the DNA chain of the pathological cells were presented.

## 2. Results and Discussion

### 2.1. Chemistry

**Figure 1:**
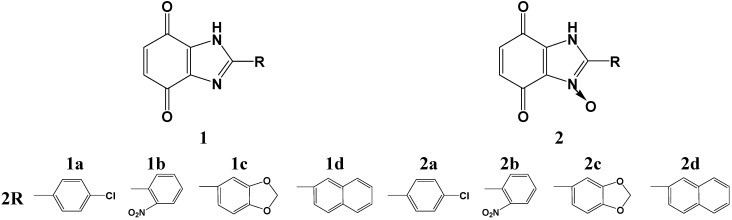
Structural formulae of benzimidazole-4,7-dione derivatives. **a**: *p*-chlorophenyl, **b**: *o*-nitrophenyl, **c**: piperonyl, **d**: naphthyl.

The structures of benzimidazole-4,7-dione derivatives **1** and *N*-oxide benzimidazole-4,7-dione derivatives **2** are shown in Figure 1. The cyclocondensation of a diamine with aldehydes was performed according to a known method described in the literature [17,19]. We worked out conditions for obtaining benzimidazole-4,7-dione derivatives **1a**–**d** and *N*-oxide benzimidazole-4,7-dione derivatives **2a**–**d** as we described earlier [20]. The structures of the benzimidazole derivatives were established by X-ray crystal structural analysis [12]. 

### 2.2. Biological Activities

The human lung adenocarcinoma A549 line and human malignant melanoma WM115 line were used for investigating *in vitro* the anticancer activity of the synthesized benzimidazole-4,7-dione derivatives **1**, **2**. The antiproliferative activity of the compounds was examined by the WST-1 assay after a 48 h exposure. The results were expressed as a relative number of viable cells. The effects of compounds on the cell apoptosis were determined by a caspase 3/7 activity assay. The effects of DNA damage were examined by using the EpiQuick *in situ* DNA damage assay kit (Epigentek, Farmingdale, NY, USA). Changes in cell morphology induced by compound treatment were visualized with a phase-contrast microscope. Moreover, we also evaluated the anticancer effects of the tested compounds in hypoxic cells. Tirapazamine and etoposide were used as reference drugs. 

### 2.3. Effect of Benzimidazole-4,7-diones on Cancer Cell Viability in Normoxia

The concentration-response analysis was performed to determine the compound concentrations required to inhibit the growth of cancer cells by 50% (IC_50_) after the incubation for 48 h. A wide range of concentrations, from 1 μM to 500 μM, were used to test the synthesized compounds. The relative numbers of live cells after treatment were evaluated by the WST-1 assay.

**Figure 2:**
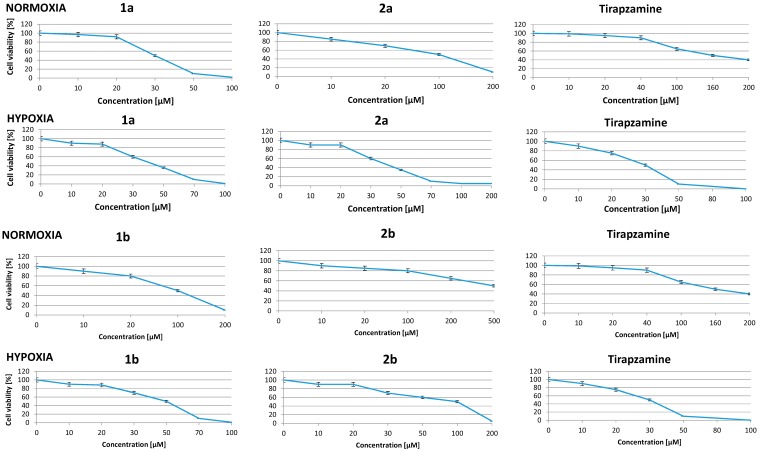
Results of the activity of some benzimidazole-4,7-diones on cancer cell viability.

Four out of eight tested compounds showed IC_50_ values below or near 100 μM in cells cultured under normoxic conditions (Table 2 compounds **1a**, **1b**, **1c**, **2c**). To compare, under the identical experimental conditions 166 μM tirapazamine was necessary in order to inhibit the 50% growth of A549 cells. Compound **1a**, with an IC_50_ value of 30.2 ± 1.2 μM, was found to have the highest anticancer activity. The culture of A459 cells in the presence of compound **1a** at a concentration *c.a.* 40 μM caused approximately a 30% inhibition of cells growth compared to control cells. However, the treatment of cells with compound **2a** at the same concentration resulted in increased cell viability by 100% (Figure 2). These results demonstrated a much higher effectiveness of compound **1a** compared to compound **2a** at a low concentration (40 μM). A lower effect on the cell growth inhibition was observed after treatment with benzimidazole-4,7-dione derivatives **1d** and **2b** (IC_50_ values 479.5 ± 3.6 μM and 500.6 ± 2.7 μM, respectively). Other tested benzimidazole-4,7-diones were less effective, but still showed good IC_50_ values over the 79.5–252 μM range and some of them (compounds **2c**, **1c**, **1b**, **2a**) showed promising IC_50_ values over the 79.5–115 μM range (Table 2).

**Table 2:** *In vitro* growth inhibition of selected tumor cell lines by some benzimidazole-4,7-diones.

Compounds		IC_50_ [μM]		Differential Cytotoxicity O/H
		Normoxia (O)	Hypoxia (H)	
**1a**	A549	30.2 ± 1.2	36.1 ± 1.2	0.83
	WM 115	26.4 ± 1.6	32.5 ± 1.2	0.81
**1b**	A549	100.0 ± 1.8	47.4 ± 1.1	2.13
	WM 115	101.0 ± 1.2	45.8 ± 1.8	2.20
**1c**	A549	80.9 ± 1.9	51.2 ± 1.5	1.58
	WM 115	77.9 ± 1.3	48.5 ± 1.3	1.60
**1d**	A549	479.5 ± 3.6	232.4 ± 1.4	2.06
	WM 115	399.5 ± 2.2	229.2 ± 0.4	1.74
**2a**	A549	115.7 ± 1.9	35.0 ± 1.6	3.28
	WM 115	105.0 ± 0.7	33.0 ± 1.9	3.18
**2b**	A549	500.6 ± 1.2	116.0 ± 0.8	4.31
	WM 115	490.6 ± 1.9	115.0 ± 1.2	4.26
**2c**	A549	79.5 ± 1.9	44.0 ± 2.5	1.80
	WM 115	65.5 ± 1.3	40.0 ± 2.0	1.63
**2d**	A549	252 ± 2.5	96.8 ± 1.9	2.59
	WM 115	189 ± 1.5	95.2 ± 2.1	1.98
**T**	A549	166.2 ± 1.6	36.0 ± 1.2	4.61
	WM 115	156.2 ± 1.6	34.0 ± 1.2	4.59

WST-1 assay was used to determine the inhibition of the cell growth. After a 48 h incubation with the tested compounds. IC_50_ values (concentration of the tested compounds causing. 50% inhibition of the cell growth compared to control cells) were calculated and expressed as the mean ± SD, n = 3.

The results showed that benzimidazole-4,7-dione derivatives which have a chlorophenyl (compounds **1a**, **2a**) or piperonyl substituent (compounds **1c**, **2c**) inhibited the growth of A549 cells more potently than analogous benzimidazole-4,7-dione derivatives which contain nitrophenyl (compounds **1b**, **2b**) or naphthyl (compounds **1d**, **2d**) ones. This relationship based on the presence of appropriate substituents in the benzimidazole ring appears in all our latest research on the approach of using two groups of potential bioreductive agents: benzimidazoles and benzimidazole-4,7-diones [12]. 

Normoxic and hypoxic A549 cells were cultured in the presence of different compounds at indicated concentrations. Cell viability was determined by WST-1 assay after a 48 h treatment. Data are expressed as a mean-SD, n = 3.

### 2.4. Effect of New Compounds on Viability of Hypoxic Cells

Hypoxia characterises the tumor microenvironment which forms the new target of novel potential anticancer substances that possess an active bioreductive mechanism. For these reasons we also evaluated the effect of our compounds on hypoxic cancer cells. A549 and WM115 cells were exposed to hypoxia (1% O_2_) for 24 h before treatment and cells were maintained under hypoxic conditions during culture in the presence of the compounds. 

As shown in Table 2, the most active agent among the benzimidazole-4,7-dione series **1**–**2** in hypoxic conditions was compounds **2b**. Under hypoxic conditions, the cell viability was significantly altered when cells were cultured in the presence of compounds **1a**, **1b**, **2a**, and **2b**. The exposure of hypoxic cells to compounds **1b** and **1d**, **2c** decreased the cell survival by 50%. Moreover, compound **2a** acted more effectively in hypoxic ce1ls in comparison to normoxic cells with IC_50_ *ca.* 35 μM and 115 μM, respectively. An identical activity is observed with compound **2d** as well. In contrast, compound **1a** showed a similar, significant anticancer effect in hypoxic and normoxic cells with any doses tested (Table 2, Figure 2). 

### 2.5. Effect of Compounds on Cell Apoptosis

**Figure 3:**
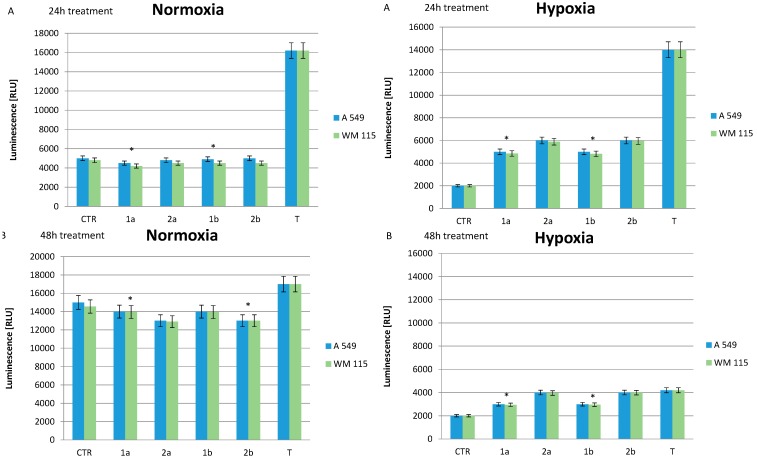
Influences of some benzimidazole-4,7-diones and tirapazamine on cell apoptosis in normoxic and hypoxic conditions.

Based on differential cytotoxicity O/H values, we selected compounds **1a**, **2a**, **1b** and **2b** for further biological evaluation. To evaluate if the inhibition of the cell growth in response to these compounds was due to the induction of apoptosis, caspase 3/7 activity was measured. The apoptosis assay was performed in normoxic and hypoxic A549 and WM115 cells exposed to selected compounds at concentrations within the IC_50_ range for different periods of time. The treatment of normoxic cells with compounds **1a**, **2a**, **1b** and **2b** did not increase caspase 3/7 activity over the control cell level either after a 24 h or 48 h exposure, but hypoxia conditions provided proofs of the increase of the apoptosis cells death after the 24 h and 48 h exposures. The tested compounds showed a higher level of caspase 3/7 activity than in the control samples during both experiments. Similarly, the treatment of normoxic and hypoxic cells with tirapazamine for 24 h resulted in the increased caspase 3/7 activity by 4-fold and 7-fold compared to control cells for each condition, respectively (Figure 3). Moreover, our results demonstrated that the culture of control cells in hypoxia decreased caspase 3/7 activity approximately 2-fold (Figure 3A) and 7-fold (Figure 3B) compared to control normoxic cells. These results showed that hypoxia generally contributes to the increased cancer cell survival by attenuation of cell apoptosis. This thesis suggests that cells A549 can adapt to hypoxia conditions. The screening test of caspase-dependent apoptosis of the tested compounds for a 48 h hypoxic and normoxic exposure showed promoting apoptotic cell death in relation to necrotic death. 

### 2.6. Effect of Compounds on DNA Damage in Normoxic and Hypoxic Cancer Cells

Phosphorylation of histone H2AX is one of the earliest chromatin modification events in response to DNA damage and can be used as a sensitive marker for such damage. To evaluate the effects of the tested compounds on DNA damage, we measured phosphorylation of H2AX at Ser139 in normoxic and hypoxic A549 and WM115 cells exposed to the selected compounds **1**–**2** and tirapazamine at the IC_50_ concentration range for 4 h.

Normoxic (**A**: 24 h, **B**: 48 h) and hypoxic (A: 24 h, B: 48 h) A549 and WM115 cells were cultured in the presence or absence (CTR, control) of the selected compounds and tirapazamine (T) as a reference compound at indicated concentrations. Cell apoptosis was determined by caspase 3/7 activity assay after the 24 h and 48 h treatments. Caspase activity was measured as a luminescence light unit [RLU] and expressed as a mean ± SD, n = 3, * *p* < 0.05.

**Figure 4:**
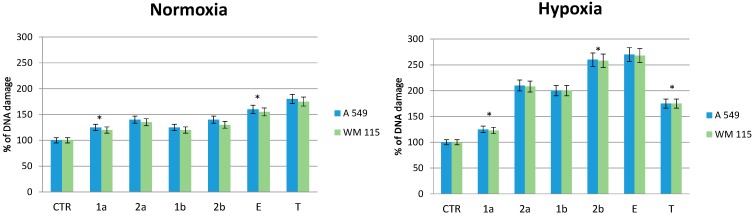
Effect of some benzimidazole-4,7-diones on DNA damage in normoxic and hypoxic A549 and WM 115 cells.

We observed a slight increase of DNA damage when the cells were cultured in the presence of the tested compounds under normoxic conditions (Figure 4 normoxia). However, tirapazamine treatment induced DNA damage by approximately 80% over control normoxic cells (Figure 4 normoxia).

In contrast to normoxic cells, the treatment of hypoxic cancer cells with compounds **2b**, **1b** and **2a**, **1a** resulted in increased phosphorylation levels of H2AX (Figure 4 hypoxia). Compound **2b** was the most potent and increased DNA damage in hypoxic cells by ~2.7 fold compared to control cells. The treatment of hypoxic cells with compounds **1b** and **2a** induced DNA damage at similar levels (~2.0 fold). In contrast, compound **1a** had a slight effect on H2AX phosphorylation. The exposure of hypoxic cells to tirapazamine also increased ~1.8 fold DNA damage as compared to control cells (Figure 4 hypoxia). 

In addition, we used etoposide, an anticancer drug known to induce DNA breakage, as positive control. Etoposide increased the levels of H2AX phosphorylation ~1.5 fold and ~2.5 fold compared to control samples in normoxic and hypoxic cells, respectively. 

Our results showed that tested compounds **2b**, **2a** and **1b** at IC_50_ range concentrations caused DNA more damage in hypoxic cancer cells than in normoxic cells. In contrast, compound **1a** does not possess any significant effect on H2AX phosphorylation level, either in normoxic or hypoxic cells. Moreover, tirapazamine and etoposide treatment increased DNA damage in normoxic as well as in hypoxic A549 and WM115 cells.

The DNA chain destruction of tumor cells. The cells samples with the tested compounds and tirapazamine (T) and etoposide (E) as reference compounds were maintained at IC50 [µM] concentrations (Table 2). The control sample (CTR) included A549 or WM115 cells without the tested substances. Statistical data: mean and s.d. of experiments are presented, n = 3, * *p* < 0.05.

## 3. Experimental Section

### 3.1. Biochemistry Experiment Procedures

#### 3.1.1. Cell Culture

A549 cells, from a human lung adenocarcinoma A549 cell line purchased from the Health Protection Agency Culture Collections (ECACC, Salisbury, UK), were cultured in F12K medium (HyClone, Loughborough, UK) supplemented with 10% heat-inactivated fetal bovine serum (FBS), penicillin (10,000 U/mL) and streptomycin (10,000 μg/mL) in 5% CO_2_ at 37 °C. Hypoxic cells were created by culture of A549 cells in a hypoxic incubator in 1% O_2_ and 5% CO_2_ at 37 °C for 24 h before treatment. 

WM115 cells, from a human malignant melanoma WM115 cell line purchased from the Health Protection Agency Culture Collections, were cultured in DMEM medium (HyClone) stabilized l-glutamine and supplemented with 10% heat-inactivated FBS and gentamycin 5 µg/mL, air 5% CO_2_ at 37 °C. Hypoxic cells were created by culture of WM115 cells in the hypoxic incubator in 1% O_2_ and 5% CO_2_ at 37 °C for 24 h before treatment. 

#### 3.1.2. DNA Damage Assay

The effect of compounds on DNA damage was determined by measuring phosphorylation of histone H2AX on serine 139 using the EpiQuick *in situ* DNA damage assay kit. A549 or WM115 cells were seeded in 96-well plates at a density of 5000 cells/well and cultured in normoxic or hypoxic conditions for 24 h before treatment. Next, cells were treated with vehicle or indicated compounds at concentrations within the IC50 range and culturing was continued under the same conditions. DNA damage in normoxic and hypoxic cells was measured after a 4 h incubation with the tested compounds. After this time, cells were fixed and assay was performed according the manufacturer’s protocol. The amount of DNA damage was proportional to the intensity of color development. The absorbance was measured at 430 nm using Synergy H1 plate reader (BioTek, Winooski, VT, USA). The % of DNA damage was calculated by OD treated sample/OD control × 100%, where OD treated samples is the absorbance of the cells treated with compounds and OD control is the absorbance of control cells treated with vehicle.

#### 3.1.3. Cell Viability/Cytotoxicity Assay

To determine the anticancer activity of the analyzed compounds, we evaluated the cell viability using WST-1 assay (Millipore, Winooski, VT, USA) according to the manufacturer’s instruction. The assay is based on the cleavage of the tetrazolium salt WST-1 to formazan resulting from the cellular mitochondrial dehydrogenases. The amount of formazan dye formed directly correlates to the number of live cells in the culture.

A549 or WM115 cells were seeded in 96-well plates at a density of 5000 cells/well and cultured in normoxic conditions. To investigate the effect of compounds on hypoxic cancer cells, A549 or WM115 cells were exposed to hypoxia (1% O_2_) for 24 h before treatment. The stock solution of the tested compounds was prepared in DMSO and diluted in complete medium to give a final concentration in the 500 M to 1 M range. Normoxic and hypoxic cells were treated with different concentrations of the tested compounds or vehicle (0.2% DMSO) for control cells. The cell viability was assessed after a 48 h incubation with compounds under normoxic or hypoxic conditions. Briefly, WST-1 reagent was added to the cells and the absorbance was determined at 440 nm using a microplate reader (Synergy H1, Bio-Tek) after a 3 h incubation at 37 °C. The percentage (%) of cell viability related to the control cells was calculated by [A] test/[A] control × 100, where [A] test is the absorbance of the cells treated with compounds and [A] control is the absorbance of the control cells. IC_50_ values (concentration of the tested compounds required to reduce cell density to 50%) were calculated by concentration-response curve fitting using a Microsoft Excel-based analytic method. 

#### 3.1.4. Apoptosis Assay

The effect of compounds on cell apoptosis was determined using the Caspase Glo 3/7 assay (Promega, Mannheim, Germany) according to the manufacturer’s instruction. The assay is based on the measurement of caspase-3/7 activity via the proluminescent substrate containing Z-DEVD-aminoluciferin (DEVD). Following caspase cleavage, a substrate for luciferase is released resulting in the luciferase reaction and the production of a luminescent signal. Tumor cells were seeded in white 96-well plates at a density of 5000 cells/well and cultured under normoxic or hypoxic conditions for 24 h before treatment with vehicle or selected compounds. Caspase 3/7 activity in normoxic and hypoxic cells was measured after the 4 h, 24 h and 48 h incubation with the tested compounds. Luminescence values were measured by a microplate reader (Synergy H1, Bio-Tek) at 135 gain.

#### 3.1.5. Cell Morphology

The effects of the tested compounds at normoxia and hypoxia on cell morphology after a 48 h treatment were evaluated with a phase-contrast microscope (OptaTech, Warsaw, Poland). 

#### 3.1.6. Statistical Analysis of the Data

The results are expressed as mean ± SD. Statistical analysis was made using Student’s *t*-test. *p* < 0.05 was considered significant.

## 4. Conclusions

**Figure 5:**
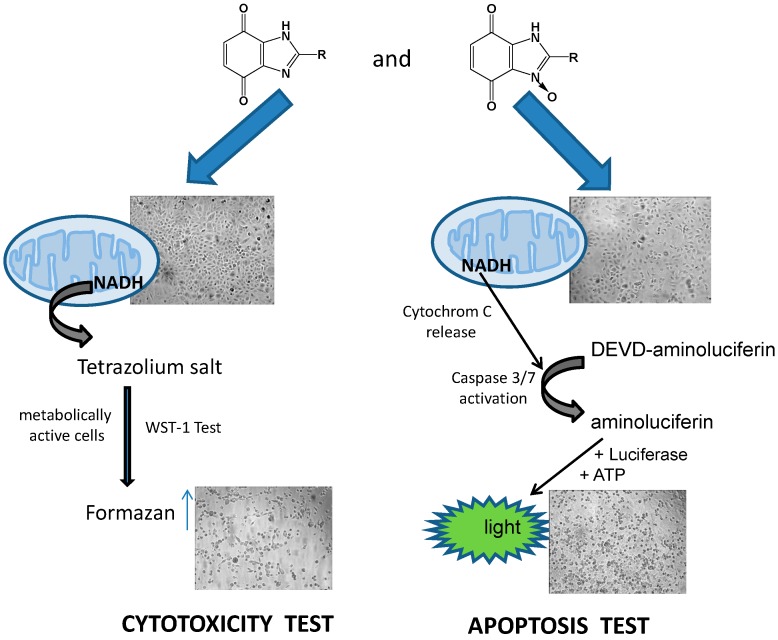
*In vitro* results of the cytotoxic and apoptosis activity of the tested substances.

The work developed in this biochemical investigation allowed us to reach some conclusions about the biological effects of the derivatives of benzimidazole-4,7-dione and their analogous *N*-oxide benzimidazole-4,7-dione derivatives. Due to the previously performed cytotoxicity test on A549 line which provided satisfactory results, we continued our research of the activity on the WM115 cell line to support and broaden the spectrum of the study. To examine and compare the activity of some heterocyclic compounds, WST-1 test, caspase 3/7 test and DNA destruction tests were performed on two tumor cell lines (Figure 5).

The morphology (×100 magnification) of A549 cells after exposure to compounds: **1a** in the normoxia cytotoxic test and **2b** in the hypoxia apoptosis test on A549 tumor cells.

The established WST-1 test allowed us to study the potential of the inhibition of the cell growth in normoxic and hypoxic A549 or WM115 cells. Our screening tests showed that compound **2b** possesses the best antiproliferation features in correlation to normoxia/hypoxia and this point suggests its potential usefulness as a selective agent for hypoxic tumor cells. Other compounds like **2a**, **2d**, **1b**, **1d** were also characterized as potential cytotoxic agents. Compound **1a** was the most potent in normoxic conditions as well as in hypoxia conditions and this result suggests that it is not selective to pathological unoxidized tumor cells. 

The applied caspase 3/7 dependent test proved that all tested compounds can promote apoptosis cells death under normoxia conditions at a level similar to the control sample. It means that their cytotoxic species do not disrupt the programmed, natural apoptotic cell death pathway. Contrary to normoxia, hypoxia leads to tumor cell death by apoptosis more than in the control sample. This suggests their stronger cytotoxic activity in the first state of the tumor development when the pathological mechanism of growing vascular cells in it is very weak. The DNA destruction test used showed that the most potent compound with regards to genetic material damage in the pathological cells is compound **2b**. Generally, all tested substances can induce DNA breaks, but their activity in hypoxia is stronger, especially when we consider compounds **2a** and **2b**. Probably, their advantage depends on two specific groups in their structure: chlorophenyl or nitrophenyl group and the *N*-oxide bond. 
